# Analysis of 121 cases of osteosarcoma of jaws reported in the literature over the past two decades: a scoping review

**DOI:** 10.1016/j.jbo.2026.100763

**Published:** 2026-04-17

**Authors:** Aisha A.H. Al-Jamaei, Sahar A.O. Al-Qayadhi, Marco N. Helder, Prasanna Sirinivas Deshpande, R.V. Subramanyam, Tymour Forouzanfar, Jan G.A.M. de Visscher

**Affiliations:** a Department of Oral and Maxillofacial Surgery, Amsterdam UMC, Amsterdam, the Netherlands; b Department of Oral Medicine, Periodontics, Diagnostics, and Oral Radiology, Faculty of Dentistry, Sanaá University, Sanaá, Yemen; c Department of Oral Surgery and Oral Medicine, Al-Razi Univeristy, Sanaá, Yemen; d Ajman Specialized Dental Center, Emirates Health Services, Ajman, United Arab Emirates; e Department of Oral Medicine and Radiology, JSS Dental College & Hospital, JSS Academy of Higher Education and Research, Mysuru, Karnataka, India; f Department of Oral Pathology, K M Shah Dental College and Hospital, Sumandeep Vidyapeeth, Vadodara, India; g Department of Oral and Maxillofacial Surgery, Leiden University, Leiden, the Netherlands

**Keywords:** Jaws, Osteosarcoma, Survey case reports, Surgery, Radiotherapy, Chemotherapy, Outcome

## Abstract

Osteosarcomas of the Jaw (OSJ) are rare malignant tumors with unique characteristics compared to those in long bones. This study aimed to describe and analyze reported cases of OSJ published within the past two decades with emphasis on demographic features, clinical presentation, radiological findings and treatment outcomes. A comprehensive search of four databases, including PubMed, Embase, Google Scholar, and Scopus, was conducted between 2003 and 2025 to identify publications reported cases of OSJ. There were 121 cases of OSJ with 84.3 % adult (median age: 35 years), and 15.7 % pediatric patients (median age: 13 years). Most cases were reported from Asia (66.9 %). Of all cases, 55.4 % occurred in the mandible. Approximately 65 % presented as painless swelling, 67.7 % (65/90) were smaller than 5 cm, and 53.3 % (31/55) exhibited paresthesia. Radiological features showed 18.8 % as radiolucent, 34.2 % as radiopaque, and 47.0 % as mixed. In 7 patients (5.8 %), OSJ occurred within fibro-osseous lesions, four of which were ossifying fibromas. OSJ presented as a periapical lesion in 4 patients (3.3 %) and developed during pregnancy in 4 patients (3.3 %). For treatment, 38.8 % underwent surgery alone, 34.7 % received surgery combined with chemotherapy, and 26.4 % received other modalities. Among 90 cases with follow-up data, 19 individuals (21.1 %) experienced local recurrence and 12 (14.3 %) developed distant metastasis**.** Surgical treatment showed the highest survival rates [pooled mean (standard error) = 54.2 (4.1) months]. Local recurrence and treatment approaches, particularly those integrating surgery with chemoradiation or alternative therapies, were associated with a significant reduced 5-year overall survival rate.

## Highlights

• Osteosarcoma of jaws can present in various clinical forms, including resembling periapical lesions.
• Patients with fibro-osseous lesions should receive vigilant monitoring for potential malignant transformation.
• Recurrence is the primary factor associated with poor survival.
• Surgical resection demonstrated the best survival outcomes.
• Complete patient narratives in published case reports would strengthen documentation and analysis, improving clinical practice.

## 1 Introduction

Osteosarcoma (OS), also known as osteogenic sarcoma, is the most common primary malignant bone tumor. This condition constitutes approximately 20 % of all primary malignant bone tumors, with an established incidence of 3.4 per million individuals [1], [2]. Studies have shown that OS stands as the predominant form of primary malignant bone tumor among adolescent patients 3,4,5. It may develop in all bones, with the highest prevalence observed in the metaphysis of long tubular bones [3] but rare in the spine, pelvis, and sacrum areas 6. Craniofacial OS includes the skull bones and accounts for 6.5–7 % of all OS cases and rank as the fourth most frequent site for osteosarcoma, following the femur, tibia, and humerus [4]. Its prevalence is approximately 0.7 per million individuals. Most craniofacial OS affect the maxillary and mandibular region [5], [6]. The predominant clinical presentations involves patients exhibiting a solitary lesion [7]. OS presents in various histological subtypes, depending on the predominant cellular component. The most common types are osteoblastic, chondroblastic, and fibroblastic OS. Telangiectatic, giant cell rich, epithelioid, small cell, and undifferentiated pleomorphic sarcomas are less common [8]. OS is characterized by its high metastatic potential and presence of cancerous cells capable of producing osteoid, chondroid matrix, or fibrotic connective tissue [9].

The etiology of OS is unknown in primary OS. In terms of genetic alterations, no specific recurring alterations have been identified so far, though some tumor suppressor genes and proto-oncogenes, such as TP53, RB1, and Myc, have been noted [10]. In contrast, secondary OS appears to be related to certain genetic syndromes, radiation exposure, and preexisting bone lesions [11], [12]. OS of the jaws (OSJ) exhibit distinct demographic characteristics when compared to OS of long bone (OSLB), particularly in terms of a later age of onset and more favorable prognostic outcomes [13], [14]. A recent review has highlighted the differences in microenvironment biology between OSJ and OSLB, particularly in the immune environment [15]. The expression of cytotoxic T lymphocyte-associated antigen 4 (CTLA-4) and programmed cell death protein 1 (PD-1) in OSLB has been found, and the use of their blockers is currently under phase I/II clinical trials [16], [17]. However, investigations concerning the immune profiles of OSJ are very limited and exploring the expressions of CD4, CD8, PD-1, and CTLA-4 in OSJ demonstrated minimal cellular expression, with no impact on clinical features [16], [18].

Due to the relatively rare prevalence of OSJ, and consequently limited reported case series, the current understanding of the demographic characteristics, clinical, radiological and histological features, treatment modalities and outcome of treatment remains constrained. A practical approach to gaining more insight in these various aspects of OSJ is through collecting and combining case reports and case series, which may provide an avenue to delve into its various aspects. The aim of this review was to compile and analyze case reports of OSJ published within the past two decades, with a focus on describing clinical presentations and management approaches.

## 2 Materials and methods

The methodology for this scoping review adhered to Arksey and O'Malley's established framework [19], in conjunction with the Preferred Reporting Items for Systematic Reviews and Meta-analysis (PRISMA) checklist [20].

Ethical approval was not necessary for this secondary analysis of previously published data.

Following the above framework and guidelines, five stages were applied:

Identifying research question

“What are the clinical characteristics, treatment modalities and 5-year survival outcomes of OSJ based on systematic analysis for published case reports”

### 2.1 Search strategy

A search of reported cases of OS occurring only in the jaws (OSJ) was carried out across PubMed, Embase, and Scopus, using a combination of the following terms: osteosarcoma, osteogenic sarcoma, jaws, maxilla, mandible and case report. These results were supplemented with a manual search of the reference lists of relevant studies to identify additional important citations. The research included English-language papers that were published spanning the past two decades (2003–2025) and data were updated in December 2025.

### 2.2 Study selection

Two authors (AAA, SAA) scrutinized/analyzed the retrieved titles and abstracts, and irrelevant studies were excluded. Full-text articles were assessed against prespecified eligibility criteria. The inclusion criteria were restricted to studies of OSJ that provided a clear and well-documented histological diagnosis, accompanied by detailed information regarding the treatment approach employed. Studies that reported adequate clinical, radiographic, and follow-up data. Case series with maximum of 7 cases were included, provided they contained sufficient data on each individual case. Exclusion criteria included reports that mentioned osteosarcoma without definitive histological subtypes. Studies with missing data for tumor margin, follow-up, recurrence, and survival combined. Studies focused exclusively on immunohistochemical analyses, radiological imaging, genetic expression profiles, cytological assessments, in vitro experiments, OSJ cases resulting from metastasis of OSLB, and review papers. This approach ensured that only the most relevant studies were included in the analysis, thereby maintaining the rigor and accuracy of the research findings.

### 2.3 Data charting and reporting

The two authors independently extracted the data using a specially designed table data format template and disagreements were resolved through mutual consultation and discussion. Information extracted from the full-text screening of included articles were categorized and included: first author, year of publication, country, patient’s gender and age with a separate group of boys and girls under the age of 17 year; clinical features including location OSJ (maxilla, mandible), type of swelling (painless or painful), tumor size (categorized into ≤ 5 cm and > 5 cm) [21], loss of sensation (no/yes), tooth mobility, radiographic features including radiolucent, radio-opaque or mixed pattern, sunburst aspect (no/yes), tooth root resorption and widening of the periodontal ligament, histopathological diagnosis, associated diseases or syndromes, treatment modality, duration of follow-up, outcome including survival, local recurrence and distant metastasis. We organized the extracted data into a distinct table and a flowchart that highlighted the significant outcomes, all of which are presented in the results section. In accordance with established guidelines for scoping reviews, we did not conduct methodological quality assessments of individual studies, as this is not a requisite component of this review type [22].

### 2.4 Data analysis

All analyses were made using the SPSS statistical package Version 28 (IBM Inc.). The median and percentages were used for descriptive statistics. The Kolmogorov-Smirnov test checked the normal distribution. To use all data sets without excluding those with missing data, the missing data patterns was first examined and confirmed that the data were missing at random (MAR). Multiple imputation techniques were then applied to handle missing values and preserve the integrity of the statistical analysis. Local recurrence was defined as tumor recurrence identified at the original surgical site. Overall survival was defined as the interval between the date of surgery and death, or censoring due to loss of follow-up. The Kaplan-Meier method was used to estimate pooled mean (standard error, SE) 5-year overall survival, and the log-rank test was applied to assess differences between groups. We did not use the median survival rate because Rubin's rule calculates the pooled mean for imputed data. Since SPSS program can not generate plot for pooled mean overall survival for imputed data, we used Python program https://www.python.org. Multivariable cox proportional hazards regression was performed for recurrence and 5-year survival, focusing on independent variables that showed *P-value* of ≤ 0.2 in univariable analysis. Results are reported as adjusted hazard ratios (aHR) with 95 % confidence intervals. A significance level of P < 0.05 was considered in this study.

## 3 Results

### 3.1 Literature search

**Fig. 1:**
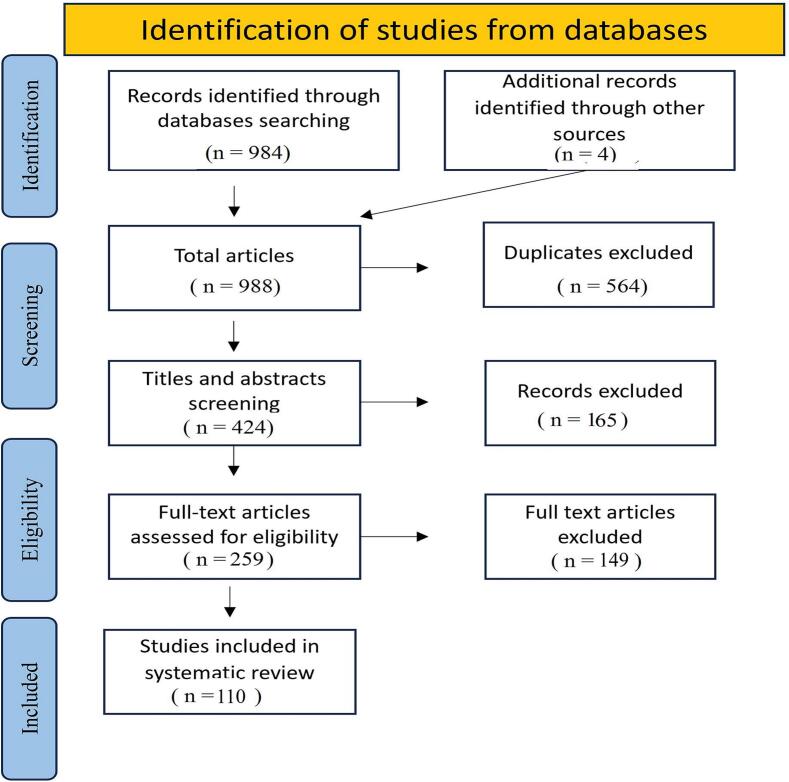
Flow chart depicting the selection process of the included studies.

The electronic search across the four different databases initially retrieved 984 articles. Four articles were identified manually from the reference lists of those articles, making the total number 988 articles. After the initial retrieval, duplicate records were eliminated resulting in 424 articles. Of these, 156 articles were excluded based on the title and abstract screening due to non-OSJ jaw lesion locations or case report status. The full text of the remaining 259 articles was thoroughly assessed against a set of prespecified eligibility criteria to ensure relevance and quality. This meticulous screening process ultimately resulted in identification of 110 articles that were appropriate for data extraction and analysis. (See Fig. 1)

### 3.2 Description of the studies and analyses

**Fig. 2:**
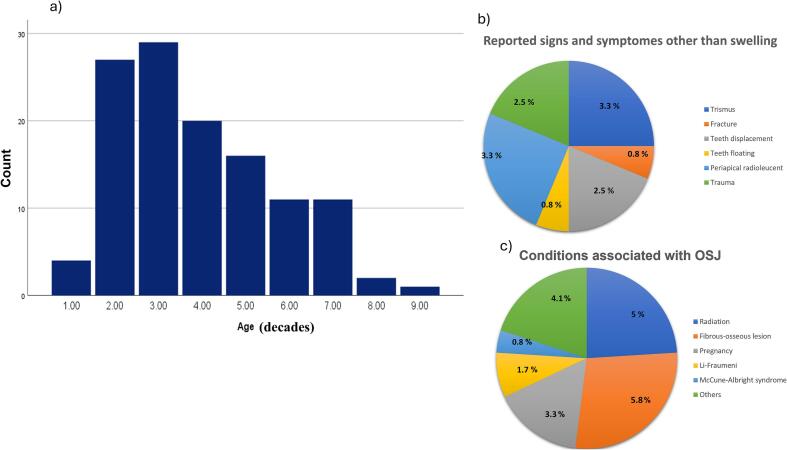
A) distribution of OSJs according to age in decades. b) charts illustrating the frequency of the diverse clinical presentations of OSJs beyond swelling. c) chart showing medical conditions associated with OSJs.

A total of 110 articles on OSJ were included in this study, encompassing 121 cases. Table 1 provides an overview of the demographic, clinical, radiographical, histopathological and treatment variables that were assessed and the numbers mentioned with percentages. When a parameter could not be assessed, this was indicated with “not mentioned” (NM), and excluded from the calculation of known parameter percentages. No gender predilection was noted: the distribution was 42.1 % for both males and females, of which 7.4 % were boys and 8.3 % girls. Fig. 2a displays the distribution of OSJ by age in decades, showing the highest prevalence in the third decade followed by the second and fifth decades. The median age of all patients was 31.0 years (range 4.5–90) and for boys and girls (13) years (range 4.5–17). Most reports of OSJ were reported from Asia (66.9 %).

**Table 1:** Demographic and clinical features of jaws osteosarcoma described in the literature (n = 121 cases).

Variables		Frequency (%)	Variable		Frequency (%)
General features			**Clinical features**		
Gender			Swelling		
Adult Pediatric	Male	51 (42.1)		Painful	42 (35.0)
	Female	51 (42.1)		Painless	78 (65.0)
	Boy	9 (7.4)		NM	1
	Girl	10 (8.3)			
Age (Years), Median	Adult	35.0 (18–90)	Lymph nodes enlargement	Yes	18 (25.0)
(Min-Max)	Pediatric	13.0 (4.5–17)		No NM	
Continents	Asia	81 (66.9)	Tooth mobility	Yes	18 (41.9)
	Europe	11 (9.1)		No	25 (58.9)
	Africa	7 (5.8)		NM	78
	America	21 (17.4)			
	Ocean	1 (0.8)			
Types	Primary	105 (86.8)	Loss of sensation	Yes	31 (56.4)
	Secondary	16 (13.2)	(Paresthesia)	No	24 (43.6)
				NM	66
Site	Right maxilla	23 (19.0)	**Radiographic features**		
	Left maxilla	23 (19.0)		Radiolucent	40 (34.2)
	Middle maxilla	2 (1.7)		Radiopaque	22 (18.8)
	Right Mandible	33 (27.3)		Mixed	55 (47.0)
	Left mandible	30 (24.8)		NM	4
	Middle mandible	4 (3.3)			
	Synchronous**	3 (2.5)			
	Metachronous***	3 (2.5)			
Histopathology	Conventional OS*	81 (66.9)	Tooth root resorption	Yes	9 (41.0)
				No	13 (59.0)
	Telengectesia OS	3 (2.5)		NM	99
	Low grade-OS	10 (8.3)			
	Small cell OS	3 (2.5)			
	Parosteal OS	15 (12.4)			
	Periosteal OS	4 (3.3)			
	Mixed OS	5 (4.1)			
Tumor size	≤ 5 cm	65 (67.7)	Widening PDL	Yes	27 (81.8)
	>5 cm	31 (32.3)		No	6 (18.2)
	NM	25		NM	88
Margin status	Negative	65 (82.1)	Sunburst	Yes	20 (60.6)
	Positive	16 (19.9)		No	13 (39.4)
	NM	40		NM	88
Treatment≠	Surgery	47 (38.8)			
	Surgery + Chemotherapy**≠**	42 (34.7)			
	Surgery + Chemotherapy + Radiotherapy	16 (13.2)			
	Others	16 (13.2)			
Local recurrence	Yes	19 (21.1)			
	No	71 (78.9)			
	NM	31			
Distance metastasis	Yes	12 (14.3)			
	No	72 (85.7)			
	NM	37			

*Conventional OS included osteoblastic, fibroblastic, chondroblastic, epitheloid, sclerosing and giant cell types. ** Synchronous: multicentric, defined as more than one OS at presentation. ***Metachrnous:

Multicentric, second primary OS developing at least 6 months after the initial diagnosis in a different location. NM: not mentioned, ≠ all chemotherapy combined (40 patients received adjuvant, 8 received neoadjuvant, and 7 received both).

OSJ was located 55.4 % in the mandible and 39.7 %. in the maxilla. Metachronous cases (second primary osteosarcoma developing at least 6 months after the initial diagnosis in a different location) and synchronous cases (multiple primary osteosarcomas discovered simultaneously or within 6 months of initial diagnosis) each represented 2.5 % of the total 121 cases. Of OSJs, 65.0 % presented as painless swelling, 67.7 % (65/96) were less than 5 cm, 41.9 % (18/43) had affected tooth mobility, and 53.3 % (31/55) presented with loss of sensation. Furthermore, a minority of patients displayed trismus (3.3 %), tooth floating (0.8 %), tooth displacement (2.5 %) (Fig. 2b). The radiographic findings revealed that 47.0 % of the lesions had a mixed radiolucent and radiopaque aspect and 66.1 % (20/33) of the cases had a sunburst appearance. Absence or presence of widening of the periodontal ligament (PDL) and tooth root resorption was mentioned in a limited number of cases. Among assessed cases, PDL expansion occurred in 81.8 % (27/33) of patients, root resorption in 41.0 % (9/22) (Table1).

Our analysis revealed that primary osteosarcoma (OS), which develops in previously healthy bone tissue, accounted for 105 cases (86.8 %), while secondary OS, which develops in bone tissue affected by radiation exposure or underlying conditions such as fibro-osseous lesions, comprised 16 cases (13.2 %). In cases of secondary OS, radiation exposure accounted for 5.0 % (6 cases), while pre-existing conditions, particularly fibro-osseous lesions, represented 5.8 % (7 cases). Analysis of these fibro-osseous lesions revealed that they comprised three cases of fibrous dysplasia, and four cases of ossifying fibroma. Additionally, four cases (3.3 %) of OS were documented during pregnancy (Fig. 2c).

There were 66.9 % conventional OS, with osteoblastic (23.1 %), chondroblast (25.6 %), fibroblastic (6.6 %), epithelial (6.6 %), and giant cell (4.1 %) types detected. The incidence of low-grade OS and parosteal OS was 8.3 % and 12.4 % of cases, respectively. Furthermore, analysis revealed 5 cases (4.1 %) of mixed histological type.

Of the ninety cases, nineteen individuals (21.1 %) experienced local recurrence following treatment, while distance recurrence was reported in 14.3 % (12/84) (Table 1). Only 17 cases reported the time to recurrence. The median time to recurrence development based on clinical and imaging findings was 10 months, with a range of 1 month to 3 years (data not shown). Table 2 presents the results of Cox-hazard regression analysis for recurrence rate. The analysis shows that treatment pattern, particularly tumors treated with a combination of surgery and chemoradiation or other modalities, and conventional histological type as significant factors associated with increased recurrence likelihood in univariate analysis. However, multivariate regression analysis demonstrated that only treatment pattern, typically palliative in nature, remained statistically significant (P = 0.016, aHR = 4.07). A trend toward lower recurrence was observed with non-conventional histological types compared to conventional types (P = 0.060, aHR = 0.39).

**Table 2:** Univariate and multivariate Cox analysis of local recurrence and 5-year overall survival (OSJ) with demographic and clinical characteristics.

Variables	Recurrence free survival				5-year overall survival				
	HR (CI 95 %)	P-value	aHR (CI 95 %)	P-value	HR (CI 95 %)		P-value	aHR (CI 95 %)	P-value
Gender Pedeatric Adults	1 1.55 (0.34–7.01)	0.561	−−	−−	1 1.32 (0.27–6.33)		0.720	−−	
Swelling		0.278	−−	−−			0.321	−−	
Painless swelling	1				1				
Painful swelling	1.54 (0.70–3.36)				2.56 (0.64–3.80)				
Tumor size		0.332	−−		1		0.183*		0.897
≤ 5 cm	1				2.00			1	
> 5 cm	1.55 (0.63–3.83)				(0.71–5.62)			0.92 (0.29–2.92)	
Type		0.312	−−	−−			0.476	−−	−−
Primary	1				1				
Secondary	1.59 (0.64–3.93)				1.44 (0.52–3.95)				
Margin		0.639	−−	−−			0.515	−−	−−
Negative	1				1				
Positive	1.25 (0.48–3.20)				1.38 (0.51–3.75)				
Treatment		**0.006**		**0.016**			**< 0.001**		**0.048**
Surgery	1		1		1			1	
S + CH≠ S + CH + Rad Other	1.00 (0.32–23.06)		0.88 (0.28–2.79)		1.20 (0.18–7.73)			1.37 (0.19–9.73)	
	2.67 (0.92–7.73)		2.20 (0.72–6.70)		4.54 (0.85–24.05)			2.78 (0.42–18.06)	
	4.90 (1.47–16.31)								
			4.07 (1.18–14.04)		9.45 (1.83–48.79)			5.54 (0.81–37.90)	
Histopathological types		**0.037**		**0.060**			0.165*		0.569
Conventional	1		1		1			1	
Others	0.36 (0.14–0.94)		0.39 (0.14–1.04)		0.42 (0.12–1.4)			0.70 (0.21–2.35)	
Site		0.912	−−	−−			0.953	−−	−−
Maxilla	1				1				
Mandible	0.95 (0.44–2.06)				0.97 (0.38–2.44)				
Local recurrence No Yes	−−	−−	−−	−−			**0.002**		**0.036**
					1			1	
					5.86 (1.95–17.59)			4.08 (1.10–15.17)	

HR: Harazd ratio, aHR: Adjusted, CI95 %: Confidence interval 95 %, Bold indicate significant value, * Variables with P−value < 0.20 were included in multivariate regression analysis.

**Fig. 3:**
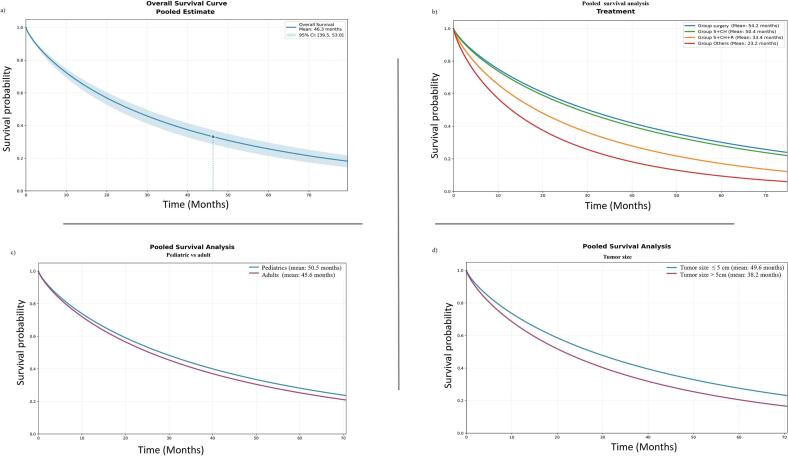
Pooled survival analysis based on different demographic and clinical variables. S + CH: Surgery + chemotherapy, S + CH + RT: Surgery + chemotherapy + radiotherapy.

Survival analysis was conducted using multiple factors to identify patterns of 5-year overall survival (Table 2 and Fig. 3). Multivariate analysis indicated that treatment modalities (P = 0.048) and local recurrence (P = 0.036) were the only factors demonstrating a statistically significant difference in survival rates across groups (Table 2). The pooled mean (SE) 5-year overall survival rates for patients with OSJ was 46.28 (3.4) months (Fig. 3a). The estimated 5-year overall survival duration was 54.2 (4.1) months for patients who received surgery alone and 50.4 (5.8) months for patients who underwent surgery and chemotherapy (Fig. 3b). Patients who underwent surgery combined with chemoradiotherapy had an estimated mean 5-year overall survival of 33.4 (6.0) months, while those who received other treatment modalities, such as radiation alone or chemotherapy alone, had a considerably lower mean survival of 23.2 (6.4) months. Tumors measuring ≤ 5 cm had a pooled mean 5-year survival rate of 49.6 (3.7) months, compared to tumors measuring > 5 cm [38.2 (6.1) months, figure 3d]. Notably, in cases with positive margins, analysis revealed a higher 5-year overall survival rate for mandible cases [46.3 (7.7) months] compared to maxilla cases [40.3 (7.7) months], though non significant (Data not shown).

## 4 Discussion

The scarcity of OSJ cases has resulted in a paucity of literature, leading to inadequate recognition of this variant till date. While several retrospective studies with large sample sizes have been conducted to provide an overview of this malignancy regarding demographic and clinical behavior, these studies were limited to specific societies. Our study, however, examines documented cases of OSJ from around the globe, focusing on the period since the 2000 s with the rise of the internet. This approach enables us to analyze a more diverse patient population and enhances the generalizability of our findings.

OSJ are known to occur in the third or fourth decades of life which is a delayed presentation in contrast to OSLB, which peaks in the second decade [23], [24]. In the present review, the average age of presentation was 31.0 years, aligning with numerous previous research findings [25], [26], [27]. However, previous study conducted on African populations with primary OSJ has reported a lower average age of 27.2 years [28]. It has been suggested that the onset of this malignancy in African populations may occur earlier, though the rate of progression remains consistent across various ethnic backgrounds [29]. Interestingly, some studies have also indicated a correlation between the age of disease onset and the specific sites of OSJ occurrence, albeit yielding inconsistent results [5], [28]. In line with the Ogunlewe *et al*. study, the current review found that the median age of presentation for patients with maxillary lesions was higher compared to those with mandibular lesions, though no significant difference was noted (data not shown) [28]. Additionally, a previous study suggested a potential link between the average age of OSJ patients and their prognosis, highlighting that the onset age of survivors averaged 27 years, while non-survivors had onset ages of 40 years and above [30]. No such significant correlation between age and survival was noted in this study. It is important to mention that around 15.7 % of individuals diagnosed with OSJ in this review were pediatric patients, averaging at 13.0 years. This data underscores that a significant fraction of those impacted by this variant of OS are young individuals. Consequently, it is crucial to take into account the distinct challenges and therapeutic regimens that pediatric patients encounter in contrast to adults.

OSJs have varied clinical presentations, however, one of the interesting finding revealed from the present review study is its resemblance with clinical and radiographic characteristics of periapical lesions. Periapical radiolucency is frequently linked with apical periodontitis caused by pulpal infection by dentists [31]. This study identified four cases of OSJ cases displayed features of periapical lesions [32], [33], [34], [35], [36], [37]. This information is extremely important for dentists since it emphasizes the importance of considering OSJ in their differential diagnosis list while treating periapical lesions.

The present study identified four reported cases of OSJ development during pregnancy [38], [39], [40], [41]. Whether this is a coincidence or reflects a true relationship between this malignancy and pregnancy remains unclear. However, hormonal variations and physiological changes during pregnancy may influence cancer onset or progression, though the precise mechanisms require further investigation [42]. A notable emphasis is placed on the unique patterns of hormone receptor expression in different types of OS. For example, Dohi *et al*. [43] highlighted elevated levels of estrogen and progesterone receptors in OS of long bones, indicating a potential hormonal role in such instances. Conversely, Dominguez-Malagón *et al*. [44] identified a lack of expression of these hormone receptors in OSJ, suggesting a possible divergence in tumor biology among different anatomical locations. Overall, the complex interplay between pregnancy, hormonal changes, and the development of OSJ warrants continued investigation.

Research has established a correlation between OS development and pre-existing bone lesions. Notably, Paget's disease, a condition frequently associated with this phenomenon, was absent in our study population [45]. This absence is likely attributable to the predominantly Asian descent of our participants. Fibrous-osseous lesions were identified in approximately seven patients (5.8 %), with one diagnosed with polyostotic fibrous dysplasia/ McCune-Albright syndrome (FD/MAS) [38], exhibiting a preponderance of female participants. This incidence appears higher than that reported by Cheng *et al*. [46], potentially due to their focus on Chinese populations, while we included diverse populations. Furthermore, a recent study from China involving 305 patients with craniofacial fibrous dysplasia lesions demonstrated malignant transformation to OSJ in 12 patients (4.0 %) [47]. A notable finding of our review was the identification of ossifying fibroma transformation to osteosarcoma in four patients, potentially representing the largest series of such cases reported to date. In a systematic review, Wagner and colleagues identified only one case of ossifying fibroma among 27 fibro-osseous lesions transforming into OSJ [48]. These findings underscore the importance of comprehensive evaluation and ongoing monitoring of pre-existing benign bone lesions, irrespective of their classification. Nonetheless, it should be mentioned that the initial diagnosis of conditions such as fibro-osseous lesions prior to OSJ might be subject to misinterpretations, especially when complete sample of biopsy are not available. This highlight inherent challenges in histopathology diagnostic interpretation.

Recurrence is common in OSJ and often leads to poor prognosis and significant treatment challenges. Kämmerer *et al*. [49] identified local recurrence as the leading cause of death in these patients. It has been reported that OS of maxilla and difficulty to achieve negative margin is the main risk of OSJ recurrence. In our analysis, we could not find such an observation. This discrepancy might be because the occurrence of recurrence in 19 out of 90 cases represents a relatively modest event rate (21.1 %). This limited number of recurrence events may explain why no statistically significant relationship was identified with important factors like tumor size and surgical margins. Statistical power to detect associations is reduced when the outcome event is infrequent, making it challenging to establish significant correlations even when clinically meaningful relationships may exist. Additionally, the follow-up duration was insufficient in many case reports. It is important to note that there is currently no consensus regarding the definition of a clear margin for OSJ, though a minimum of 3 mm on permanent histological section has been proposed [50], [51]. With respect to our data, we observed that some researchers considered a 0.5 cm margin as clean, while others only classified margins of 1–2 cm on average as free. Additionally, certain case reports indicated free margins without specifying precise measurements. We thus adhered to the authors' original categorizations of their case reports.

The case reports in this study clearly illustrated how metastases detrimentally affect the overall survival rates of individuals with OSJ. Of particular interest, our findings indicated a notably low occurrence of metastasis in OSJ (12 out of 84, 9.9 %). This aligns well with the metastatic rates reported in the literature, which range between 6 % and 21 % [25], [52]. It also corresponds with the conclusion drawn by Baumhoer *et al*., indicating that patients with OSJ developed metastases significantly less frequently (17.3 %) and later in the course (mean 26 months after diagnosis) compared to patients with OSLB [53]. Our analysis also revealed a significant difference: the mean survival rate for patients with metastasis was 31.2 ± 6.4 months, while it was 54.1 ± 2.3 months for patients without metastasis (data not shown). Likewise, a study by the DOESAK group reported that the 5-year survival rate amongst patients without metastasis was 78.3 %. If metastasis developed, the rate dropped to 19.7 % [53]. Altogether, our study underscores the relatively low incidence of metastasis in OSJ and highlights its substantial impact on patient survival. It also emphasizes that OSJ cannot be compared with the OS of long bones.

While multimodal treatment approaches have demonstrated improved survival rates in OSJ [54], our analysis found that such treatment was significantly associated with reduced 5-year overall survival. This finding may be explained by the fact that multimodal therapies are typically reserved for patients with advanced-stage tumors or more aggressive disease presentations. Furthermore, this result could indicate the complexity of tumor biology and treatment resistance, or reflect adverse effects and patients poor tolerance to combined therapeutic regimens.

The evidence regarding the efficacy of neoadjuvant and/or adjuvant chemotherapy in osteosarcoma of the jaw (OSJ) remains inconsistent [55], [56]. Our analysis revealed that the highest survival rates were observed with surgical treatment protocols. This finding is consistent with the study by Baumhoer *et al*. [53] study, which reported no survival benefits from the addition of pre-operative or post-operative chemotherapy, attributing this to bone scaffold formation by cancer cells. Additionally, two recent reviews found no benefits in incorporating chemotherapy into OSJ treatment protocols [57], [58]. Interestingly, Chen *et al*. [59] found a short-term survival benefit for adjuvant chemotherapy, but this advantage was not with long-term survival analysis. Additionally, Liang and co-authors [60] identified a survival benefit for adjuvant chemotherapy specifically for patients with positive margin and high grade OSJ. The most recently published consensus guidelines on OSJ treatment also recommend chemotherapy specifically for high-grade tumors [61]. Notably, comparative analysis of adjuvant chemotherapy versus neoadjuvant or combined treatment approaches in this cohort demonstrated that post-operative chemotherapy yielded more favorable patient outcomes. These findings underscore the ongoing debate regarding the efficacy of neoadjuvant and/or adjuvant chemotherapy in osteosarcoma of the jaws and highlight the need for additional studies to establish an optimal treatment protocol for this variant of osteosarcoma.

Incorporating radiotherapy and chemotherapy with surgery for OSJ is generally recommended in cases presenting with not-clear margins, surgical infeasibility, or local recurrence [62]. While Guadagnolo et al. advocate for this multi-modal approach, our research indicates a notable decrease in survival rates [25]. Our analysis suggests that the primary obstacle in improving survival rates through this approach is the high rate of recurrence, with 6 out of 13 cases treated with this combined modality experiencing disease reoccurrence. It is noteworthy that one study documented a transformation of osteosarcoma to rhabdomyosarcoma, potentially linked with exposure to chemoradiation [63]. Additionally, there is a possibility that OSJ may exhibit resistance to radiotherapy, comparable to OSLB, suggesting limited benefit from the inclusion of this treatment modality [23].

The main limitation of our study is that it was based on case reports, some of which were not well-documented. Indeed, our literature search identified a substantial number of case reports with missing critical data that required us to limit the scope of our study. For instance, approximately 30 reports did not clearly specify the histological types of OSJ, and other reports lacked comprehensive details regarding treatment protocols, follow-up data, occurrence of local or distant recurrence, or details of stages of the lesions. Nonetheless, we included articles with minimal missing data where feasible. Furthermore, the insufficient data prevented us from properly categorizing the lesions according to their grade classifications (low, intermediate, or high) or included clinical stage in the analysis. While case reports represent valuable resources for understanding rare diseases, journals should establish more rigorous acceptance criteria. There is a clear need for more complete patient narratives in published case reports, as thorough documentation and subsequent analysis would significantly enhance current clinical practice.

In conclusion, case reports are a valuable resource for oncology research, particularly in rare malignancies like OSJ. This study revealed that OSJ can present in various clinical forms, including resembling periapical lesions, which are pivotal for dentists to recognize. The present study also showed that OSJ occurred during pregnancy and a higher transformation rate in fibro-osseous lesions, including ossifying fibroma. The main prognostic factors for survival rates were the presence of local and distance recurrence and the use of combined and advanced treatments.

## Authors contribution

A.A.A. contributed to data collection, analysis and interpretation, and drafting the manuscript. S.A.A. contributed to data collection, analysis and interpretation, and drafting the manuscript. M.N.H. contributed to the study's conception and design, data analysis and interpretation, and revising the manuscript critically. P.S.D. contributed to data collection, analysis and interpretation, and drafting the manuscript. R.V.S. contributed to the study's conception and design, revising the manuscript critically. T.F. contributed to the study's conception and design, data analysis and interpretation, and revising the manuscript critically. J.D.V. contributed to the study's conception and design, data acquisition, analysis, and interpretation, and revising the manuscript critically. All authors read and approved the final version of the manuscript.

## CRediT authorship contribution statement

**Aisha A.H. Al-Jamaei:** Conceptualization, Data curation, Software, Writing – original draft. **Sahar A.O. Al-Qayadhi:** Data curation, Methodology, Software, Writing – original draft. **Marco N. Helder:** Conceptualization, Data curation, Methodology, Software, Supervision, Writing – review & editing. **Prasanna Sirinivas Deshpande:** Data curation, Formal analysis, Methodology, Writing – original draft. **R.V. Subramanyam:** Data curation, Formal analysis, Methodology, Software, Writing – review & editing. **Tymour Forouzanfar:** Conceptualization, Formal analysis, Methodology, Software, Supervision, Writing – review & editing. **Jan G.A.M. de Visscher:** Conceptualization, Data curation, Formal analysis, Methodology, Software, Supervision, Writing – review & editing.

## Funding

The authors declare that no funds, grants, or other support were received during the preparation of this manuscript.

## Declaration of competing interest

The authors declare that they have no known competing financial interests or personal relationships that could have appeared to influence the work reported in this paper.
