# Supplementary material for: Analysis of 121 cases of osteosarcoma of jaws reported in the literature over the past two decades: a scoping review
Source: J Bone Oncol. 2026 Apr 17;58:100763. doi: 10.1016/j.jbo.2026.100763 (PMC13199937; doi:10.1016/j.jbo.2026.100763)
Supplement: Supplementary Data 1 [file mmc1.docx]

**References for studies included in the analysis**

1. Al-Rabadi O, Alkofahi HH, Hamad R, Al-Shishani T, Al-Nawafla M. Diagnosis Dilemma of Fibroblastic Osteosarcoma: A Case Report. Cureus. 2024;16(11):e74093.
2. Al-Yahya A, BinAhmed A, Alshammari T. Maxillary osteosarcoma: two case reports and literature review. Oral Health Dent Manag. 2014;13(2):525–8.
3. Arnold JP, Smith CJ, Torres KEG, Jenzer AC. Low-grade central osteosarcoma of the mandible clinically mimicking a benign fibro-osseous lesion: A rare case report. Dent. 2020;6:1-6.
4. Abe A, Kono T, Uchida K, Takahashi Y, Kawano K. Osteosarcoma of the jaw: Report of four cases. Journal of Oral and Maxillofacial Surgery, Medicine, and Pathology. 2022;34(2):178-84.
5. Abtahi J, Ajan A. Malignant transformation of ossifying fibroma into parosteal osteosarcoma with high-grade component: presentation of an unusual case and review of the literature. The Open Dentistry Journal. 2018;12:1059-68.
6. Adwani D, Bhattacharya A, Adwani N, Adwani R, Sharma VW. Massive recurrent chondroblastic osteosarcoma of maxilla: a rare case report. J Clin Diagn Res. 2014;8(1):288-90.
7. Agrawal RR, Bhavthankar JD, Mandale MS, Patil PP. Osteosarcoma of Jaw with Varying Histomorphologic Patterns: Case Report. J Orthop Case Rep. 2017;7(1):61-4.
8. Ahmad I, Bhatt CP, Bashir I, Rathour S. High-grade osteosarcoma of the mandible: a rare tumor successfully treated with surgery and image-guided volumetric modulated arc therapy. BMJ Case Rep. 2018;2018.
9. Ahuja M, Mandal S, Mallya V, Khurana N, Meher R, Singh K. Osteosarcoma of the jaw: Primary versus secondary - A report of two cases. J Cancer Res Ther. 2023;19(7):2086-9.
10. Amer HW, Algadi HH, Hamza SA. Mandibular small cell osteosarcoma: a case report and review of literature. J Egypt Natl Canc Inst. 2023;35(1):30.
11. Angiero F, Moltrasio F, Cattoretti G, Valente MG. Clinical and histopathological profile of primary or secondary osteosarcoma of the jaws. Anticancer research. 2011;31(12):4485-9.
12. Augustine D, Murali S, Sekar B. High-grade chondrosarcoma of the mandible: A rare case report with immunohistochemical findings. Journal of Advanced Clinical and Research Insights. 2014;1(3):99-101.
13. Babazade F, Mortazavi H, Jalalian H. Bilateral metachronous osteosarcoma of the mandibular body: a case report. Chang Gung Med J. 2011;34(6 Suppl):66-9.
14. Babu H, Sharma R, Sharma VK, Aggarwal T, Rana AK. A case of recurrent chondroblastic osteosarcoma mandible. International Journal of Otorhinolaryngology and Head and Neck Surgery. 2021;7(4):698.
15. Bajpai M, Pardhe N, Chandolia B, Arora M. Osteogenic Sarcoma of Mandible. J Coll Physicians Surg Pak. 2017;27(3):S26-S7.
16. Balwani S, Tupkari J, Barpande S. Parosteal osteosarcoma of the mandible. Journal of Oral and Maxillofacial Pathology. 2006;10:10.
17. Bansal A, Bhalekar S, Shetty D. Osteosarcoma of jaw, common entity at an uncommon site: a rare case report. International Journal of Research in Orthopaedics. 2022;9:207.
18. Behere R, Lele S. Synchronous osteosarcoma of mandible. Oral Surg Oral Med Oral Pathol Oral Radiol Endod. 2009;107(5):e45-9.
19. Bhosale T, Bhate K, Bawane S, Londhe U. Low-grade central osteosarcoma of the maxilla: A diagnostic dilemma. Oral and Maxillofacial Surgery Cases. 2023;10:100339.
20. Bojan A, Christy W, Chanmougananda S, Ashokan K. Osteosarcoma of mandible: A case report and review of literature. Journal of Clinical and Diagnostic Research. 2012;6:753-7.
21. Bousdras VA, Flanagan A, Bousdras KA, Vourvachis M, Newman L, Kalavrezos N. Multiple-site osteosarcomas of the jaw in a single patient. A true case of a metachronous lesion? Int J Oral Maxillofac Surg. 2010;39(7):733-6.
22. Cabral LA, Werkman C, Brandao AA, Almeida JD(2009)Imprint cytology of osteosarcoma of the jaw: a case report.J Med Case Rep.3:9327.DOI: 10.1186/1752-1947-3-9327.
23. Carlos-Bregni R, Contreras E, Hiraki KR, Vargas PA, León JE, de Almeida OP. Epithelioid osteosarcoma of the mandible: a rare case with unusual immunoprofile. Oral Surgery, Oral Medicine, Oral Pathology, Oral Radiology, and Endodontology. 2008;105(2):e47-e52.
24. Chabchoub I, Gharbi O, Remadi S, Limem S, Trabelsi A, Hochlef M, et al. Postirradiation osteosarcoma of the maxilla: a case report and current review of literature. J Oncol. 2009;2009:876138.
25. Chakravarthi PS, Kattimani VS, Prasad LK, Satish PR. Juxtacortical osteosarcoma of the mandible: Challenges in diagnosis and management. Natl J Maxillofac Surg. 2015;6(1):127-31.
26. Costello L, Toner M, Pierse D, Stassen LFA. Osteosarcoma (osteogenic sarcoma) of the jaws presenting in general dental practice - a series of four cases. Br Dent J. 2021;230(9):583-6.
27. Cutilli T, Scarsella S, Fabio DD, Oliva A, Cargini P. High-grade chondroblastic and fibroblastic osteosarcoma of the upper jaw. Ann Maxillofac Surg. 2011;1(2):176-80.
28. Dabas SK, Menon NN, Ranjan R, Shukla H, Gurung B, Tiwari S, et al. A Rare Case Report of Osteosarcoma of Maxilla with Double Free-Flap Reconstruction. Indian J Surg Oncol. 2024;15(Suppl 1):172-8.
29. Dandagi S, Tambuwala A, Jain A, Sangle A, Kaul D. Periosteal Osteosarcoma with Diabetes Mellitus in a 20 year old female. IJDA. 2010;2(2):234.
30. De Biase A, Morciano W, Carpino F, Milella M. Aggressive chondroblastic osteosarcoma of the jawbone. Oral Oncology Extra. 2005;41(10):296-8.
31. Desai D, Pandith S, Jeergal PA, Arathi K, Saini R. Fibroblastic variant of osteosarcoma: a challenge in diagnosis & management. Open Dent J. 2010;4:211-7.
32. Dioufa N, Zhang P, Black R, Khurana J, Kuklani R. A rare case of parosteal osteosarcoma of the maxilla in a 14 year old and a review of the current literature. Oral Surgery. 2020;13.
33. Donaldson ME, Geist JR, Daley TD. Osteosarcoma of the jaws in children. Int J Paediatr Dent. 2004;14(1):54-60.
34. Evangelista K, de Faria Vasconcelos K, Teodoro AB, Cavalcanti MGP, de Mendonca EF, Watanabe S, et al. Malignant tumours mimicking periapical lesions: A report of three cases and literature review. Aust Endod J. 2022;48(3):515-21.
35. Eya M. Radiological Monitoring of the Evolution of Initially Misdiagnosed Advanced Maxillary Osteosarcoma: A Case Report. Saudi J Oral Dent Res. 2021;6(9):410-7.
36. Fang Z, Jin S, Zhang C, Wang L, He Y. Misdiagnosis of osteosarcoma as cementoblastoma from an atypical mandibular swelling: A case report. Oncol Lett. 2016;11(6):3761-5.
37. Fiori R, Vivo D, Scarano AL, D'Onofrio S, Calabria E, Giovanni S. Local invasion of jaw osteosarcoma. International Journal of Case Reports and Images (IJCRI). 2015;6(4):224-7+ viii.
38. Fu HH, Zhuang QW, He J, Wang LZ, He Y. Giant cell-rich osteosarcoma or giant cell reparative granuloma of the mandible? J Craniofac Surg. 2011;22(3):1136-9.
39. Gahan R, Rout J, Webster K. Case report: oral manifestations and radiographic features of osteosarcoma. Dent Update. 2007;34(1):52-4.
40. Garud MS, John A, Umarji HR. Osteosarcoma of The Maxilla : A Case Report. Journal of Indian Academy of Oral Medicine and Radiology. 2005;17(3).
41. George A, Mani V. Gnathic osteosarcomas: review of literature and report of two cases in maxilla. Journal of Oral and Maxillofacial Pathology. 2011;15(2):138-43.
42. Gupta S, Parikh S, Goel S. Parosteal osteosarcoma of mandible: A rare case report. J Cancer Res Ther. 2018;14(2):471-4.
43. Haefliger S, Harder D, Kovac M, Linkeschova K, Eufinger H, Baumhoer D. Osteosarcoma of the Mandible in a Patient with Florid Cemento-Osseous Dysplasia and Li-Fraumeni Syndrome: A Rare Coincidence. Head Neck Pathol. 2021;15(2):704-8.
44. Halder GC, Patsa S, Jadav RB, Ray JG. Osteosarcoma of mandible: A case report. Int J Case Rep Imag. 2015;6:280.
45. Hameed S, Anto A, Vengal M, Ramachamparambathu A, Sampath P. Osteosarcoma of Maxilla -An Unusual Entity: Case Report and Review of Literature Case Report ABSTRACT History. 2023:353-8.
46. Harazono Y, Yamashiro M, Yoshitake H, Kayamori K, Izumo T, Harada K. A case of highly suspected small cell osteosarcoma in the mandible. Journal of Oral and Maxillofacial Surgery, Medicine, and Pathology. 2015;27.
47. Ha TW, Park S, Youn MY, Kim DW, Kim HJ. Carbon-ion radiotherapy in osteosarcoma of the mandible: a case report. J Korean Assoc Oral Maxillofac Surg. 2021;47(4):315-20.
48. Hewitt KM, Ellis G, Wiggins R, Bentz BG. Parosteal osteosarcoma: case report and review of the literature. Head & Neck: Journal for the Sciences and Specialties of the Head and Neck. 2008;30(1):122-6.
49. Hoshi I, Abe R, Onodera K, Ohashi Y, Kawai T, Miyamoto I, et al. Osteosarcoma of the Mandible in an Elderly Patient. Case Rep Dent. 2022;2022:2622551.
50. Huang TC, Monsour PA, Chahoud CD. Parosteal osteosarcoma: report of a case and review of the literature. Aust Dent J. 2010;55(1):86-91.
51. Ilić MP, Kiralj A, Markov B, Mijatov I, Mijatov S, Vučković N. Li-Fraumeni syndrome: a case report. Vojnosanit Pregl. 2014;71(12):1159-62.
52. Isokane M, Sumida T, Okuhira T, Shintani S, Hamakawa H. Surface osteosarcoma: 2 case reports. Am J Otolaryngol. 2006;27(5):349-52.
53. Indermun S, Titinchi F, Alwan J, Morkel J, Nortje CJ. Osteosarcoma associated with cemento-osseous dysplasia: co-incidence or two related entities? Oral Radiol. 2024;40(4):546-54.
54. Jot K, Nayyar V, Manchanda S, Bhutia O, Mishra D. Telangiectatic osteosarcoma of maxilla in a young child. Oral Oncol. 2023;147:106605.
55. Jot K, Roychoudhury A, Bhalla AS, Mishra D. Rare case of primary giant cell rich osteosarcoma in mandible. Oral Oncol. 2022;127:105784.
56. Kalburge JV, Sahuji SK, Kalburge V, Kini Y. Osteosarcoma of mandible. J Clin Diagn Res. 2012;6(9):1597-9.
57. Kanazawa I, Yamauchi M, Yano S, Imanishi Y, Kitazawa R, Nariai Y, et al. Osteosarcoma in a pregnant patient with McCune-Albright syndrome. Bone. 2009;45(3):603-8.
58. Kaur KP, Kundu S, Chatterjee A, Pal M. Fibroblastic osteosarcoma: A perplexing entity. J Oral Maxillofac Pathol. 2023;27(3):573-8.
59. Kaveri H, Rekha K, Punnya VA. Epithelioid osteosarcoma of the maxilla: report of an unusual case. Br J Oral Maxillofac Surg. 2009;47(2):143-5.
60. Khan E, Verma S, Sivakumar N, Sharma P, Chandra S, Gupta S. Telengiectatic osteosarcoma of mandible with special emphasis on immunohistochemical and molecular characteristics: A case report with review of literature. Oral Surgery. 2023;17.
61. Kumar P, Narayan B, Urs AB, Mohanty S, Augustine J, Singh P, et al. Telangiectatic osteosarcoma of the mandible-A rare case report and an insight into differential diagnosis. Rev Esp Patol. 2024;57(3):225-9.
62. Kuo C, Kent PM. Young Adult With Osteosarcoma of the Mandible and the Challenge in Management: Review of the Pediatric and Adult Literatures. J Pediatr Hematol Oncol. 2019;41(1):21-7.
63. Kupeli S, Varan A, Akyuz C, Yucel T, Ozgen B, Onder S, et al. Maxillofacial osteosarcoma successfully treated with surgery and adjuvant chemotherapy in a child. Bratisl Lek Listy. 2012;113(11):661-3.
64. Kaimal VG, Pai A. Osteosarcoma of Mandible - A Case Report. Indian J Surg Oncol. 2024;15(3):495-8.
65. Kumaravelu C, Sathya Kumar D, Chakravarthy C, Kishore Kumar RV, Rajasekhar G, Gokul Chandra Reddy M, et al. Chondroblastic osteosarcoma of maxilla: a case report and review of literature. J Maxillofac Oral Surg. 2009;8(3):290-3
66. Li BB, Zhang JY, Gao Y. Chondroblastic osteosarcoma arising in the maxilla mimicking the radiographic and histological characteristics of cemento-osseous lesions: A case report. Mol Clin Oncol. 2017;6(5):733-6.
67. Mahmood H, Hankinson P, Andrew D, Nusrath M, Khurram SA. Radiation‐induced osteosarcoma involving the mandible—Report of a rare diagnosis. Oral Surgery. 2023;16(3):279-82.
68. Malik F, Gleysteen JP, Agarwal S. Osteosarcoma of the jaw: report of 3 cases (including the rare epithelioid variant) with review of literature. Oral Surg Oral Med Oral Pathol Oral Radiol. 2021;131(3):e71-e80.
69. Mamachan P, Dang V, Bharadwaj NS, DeSilva N, Kant P. Chondroblastic osteosarcoma-A case report and review of literature. Clin Case Rep. 2020;8(11):2097-102.
70. Madiraju GS(2021)Parosteal osteosarcoma of the mandible in a pediatric patient.Oral Oncol.117:105193.DOI: 10.1016/j.oraloncology.2021.105193.
71. Margiono EA, Andreani S. Case report of a very rare primary malignant bone tumor: Mixed type maxillary osteosarcoma. Radiol Case Rep. 2023;18(5):1680-5.
72. McGuff HS, Heim-Hall J, Holsinger FC, Jones AA, O'Dell DS, Hafemeister AC. Maxillary osteosarcoma associated with a dental implant: report of a case and review of the literature regarding implant-related sarcomas. J Am Dent Assoc. 2008;139(8):1052-9.
73. Mirmohammad Sadeghi H, Karimi A, Derakhshan S, Aminishakib P, Parchami K. Conventional osteosarcoma of the mandible: Report of a rare case. Clin Case Rep. 2021;9(9):e04843.
74. Mohanavalli S, Viswanathan M, Karthikeyan R, Gnanaguru V, Vijayabala GS, Rani LJ. Osteosarcoma of the mandible and its management: a rare case report. Surgery Case Reports. 2024;1:100009.
75. Naik LK, Shetty P, Teerthanath S, Jagadeesh HM. Telangiectatic osteosarcoma affecting the mandible. J Oral Maxillofac Pathol. 2014;18(Suppl 1):S143-6.
76. Nath J, Das A, Khanikar D, Ahmed S, Kakati K. Chondroblastic Osteosarcoma of the Maxilla with Poor Response to Neoadjuvant Chemotherapy: A Rare Case Report and Updated Review of Literature. Indian J Otolaryngol Head Neck Surg. 2023;75(4):4041-6.
77. Nthumba PM. Osteosarcoma of the jaws: a review of literature and a case report on synchronous multicentric osteosarcomas. World J Surg Oncol. 2012;10:240.
78. Omoregie OF, Osaguona A, Ogbeide E. Parosteal osteosarcoma of the maxilla: A case report and review of the literature. Nigerian Journal of Surgical Sciences. 2018;28(1):18-21.
79. Opoko U, Sabr A, Da Silva FN, Raiteb M, Regragui M, Slimani F. A case of very aggressive maxilar osteosarcome in a young subject. Advances in Oral and Maxillofacial Surgery. 2021;3:100144.
80. Pardhe ND, Jamdade A, Bajpai M, Kashyap N, Mathur N. Giant Osteosarcoma of the Mandible. J Coll Physicians Surg Pak. 2017;27(9):S117-s9.
81. Padilla RJ, Murrah VA. The spectrum of gnathic osteosarcoma: caveats for the clinician and the pathologist. Head and neck pathology. 2011;5:92-9.
82. Prabhusankar K, Karande A, Jerry J, Rishal Y. Osteosarcoma of the posterior maxilla. Journal of International Society of Preventive and Community Dentistry. 2016;6:171.
83. Puranik SR, Puranik RS, Ramdurg PK, Choudhary GR(2014)Parosteal osteosarcoma: Report of a rare juxtacortical variant of osteosarcoma affecting the maxilla.J Oral Maxillofac Pathol.18(3):432-6.DOI: 10.4103/0973-029X.151340.
84. Rajab MK, B IA, Al-Hakami HA, Al-Maghrabi H. Diagnostic Challenges in Chondroblastic Maxillary Osteosarcoma: A Case Report. Cureus. 2020;12(6):e8418.
85. Rinaggio J, Kewitt GF, McGuff HS. Epithelioid osteosarcoma presenting as a rapidly expanding maxillary mass. Head & Neck: Journal for the Sciences and Specialties of the Head and Neck. 2007;29(7):705-9.
86. Sanatkhani M, Rasekhi J, Dalirsani Z, Ghazi N. Maxillofacial Malignancy in Pregnancy; Report of Two Cases. IJBC. 2019;11(4):143-7.
87. Santos TA, Américo MG, Priante AVM, Oliveira MFd, Anbinder AL. Jaw osteosarcoma and pregnancy: a rare coexistence. Autopsy and Case Reports. 2022;12:e2021359.
88. Sasaki A, Miyashita H, Kawaida M, Kameyama K. Low-grade osteosarcoma is predominant in gnathic osteosarcomas: A report of seven cases of osteosarcoma of the jaw. Clin Exp Dent Res. 2021;7(6):1175-82.
89. Selvakumar AS, Rajalakshmi V. Small cell osteosarcoma of the maxilla. Indian J Pathol Oncol. 2017;4(4):655-7.
90. Sethi A, Rehani S, Arya K. Small cell osteosarcoma of mandible: A rare case report and review of literature. J Clin Exp Dent. 2010;2(2):e96-9.
91. Shetty SS, Dahake RN, Venkadasalapathi N, Radhakrishnan R. Giant cell rich osteosarcoma of the jaw-A rare entity and review of literature. Journal of Oral and Maxillofacial Surgery, Medicine, and Pathology. 2018;30(3):301-5.
92. Silveira HA, Coelho MC, Silva EV, Nogueira GM, Diaz KP, Oliveira-Santos C, et al. Maxillary Parosteal Osteosarcoma: Additional case Report and Literature Review of Surface Osteosarcomas. Indian J Otolaryngol Head Neck Surg. 2023;75(2):1076-80.
93. Simon D, Dominic S, Varghese KG. Juxtacortical osteogenic sarcoma of the jaws: case report and review of the literature. J Oral Maxillofac Surg. 2011;69(2):527-31.
94. Shimamoto H, Ozono K, Nakamura T, Akiyama H, Gamoh S, Tamaki J, et al. Osteosarcoma in the maxilla: a case report. Oral Radiology. 2006;22(1):37–40.
95. Sinha R, Roy Chowdhury S, Chattopadhyay P, Rajkumar K. Low-grade osteosarcoma of the mandible. Journal of maxillofacial and oral surgery. 2010;9:186-90.
96. Simpson J, Sallies M, Afrogheh AH. Exploring Paediatric Jaw Osteosarcoma: A Detailed Case Report of a 12-Year-Old Girl and Review of the Literature. Case Rep Dent. 2025;2025:7428891.
97. Silva JC, de Santana DA, Fernandes IT, Santana AM, Padilha WSM, de Aquino Xavier FC, et al. Epithelioid Osteoblastoma: Clinicopathologic Features of a Classic Case with Aggressive Behavior. Head Neck Pathol. 2025;19(1):113
98. Sue M, Oda T, Sasaki Y, Kameta A, Okada Y, Ogura I. Osteosarcoma of the Mandible: a Case Report with CT, MRI and Scintigraphy. Chin J Dent Res. 2017;20(3):169-72.
99. Sun LM, Zhang QF, Tang N, Mi XY, Qiu XS. Giant cell rich osteosarcoma of the mandible with abundant spindle cells and osteoclast-like giant cells mimicking malignancy in giant cell tumor. Int J Clin Exp Pathol. 2015;8(8):9718-22.
100. Tahmasbi-Arashlow M, Barnts KL, Nair MK, Cheng YL, Reddy LV. Radiographic manifestations of fibroblastic osteosarcoma: A diagnostic challenge. Imaging Sci Dent. 2019;49(3):235-40.
101. Upasham P, Gupta P, Bangar A. Osteosarcoma of mandible in a teenager-A case report with review of literature. 2020.
102. Urs AB, Kumar P, Augustine J, Sharma P, Narayan B. Pan-Cytokeratin Positive Fibroblastic Osteosarcoma of Jaw: An Extremely Rare Entity in a Pediatric Patient. Clin Med Insights Pediatr. 2019;13:1179556519842827.
103. Vaidya SB, Nadarajan S, Galinde JS, Bhardwaj AS. Juxtacortical osteosarcoma of mandible. Ann Maxillofac Surg. 2011;1(2):172-5.
104. Venkatesh K, Priyanka T, Rukmini NS, Bisanna J. Chondroblastic Variant of Osteosarcoma of Mandible: Report of a Rare Case. J Clin Diagn Res. 2016;10(8):ED12-4.
105. Verma RK, Gupta G, Bal A, Yadav J. Primary giant cell rich osteosarcoma of maxilla: an unusual case report. J Maxillofac Oral Surg. 2011;10(2):159-62.
106. Wang GD, Zhao YF, Liu Y, Jiang L, Jiang XZ. Periosteal osteosarcoma of the mandible: case report and review of the literature. Journal of oral and maxillofacial surgery: official journal of the American Association of Oral and Maxillofacial Surgeons. 2011;69(6):1831.
107. Yamagata K, Ishibashi-Kanno N, Bukawa H. RE: High-grade osteosarcoma arising in DCIA flap reconstruction after a prior resection of maxillar cemento-ossifying fibroma: A case report. J Stomatol Oral Maxillofac Surg. 2023;124(6S2):101636.
108. Yamamoto-Silva FP, Silva BSF, Batista AC, Mendonça EF, Pinto-Júnior DDS, Estrela C. Chondroblastic osteosarcoma mimicking periapical abscess. J Appl Oral Sci. 2017;25(4):455-61.
109. Yoon JH, Yook JI, Kim HJ, Cha IH, Kim J. Periosteal osteosarcoma of the mandible. Journal of oral and maxillofacial surgery. 2005;63(5):699-703.
110. Zhang Q, Li Y, Gao N, Huang Y, Li LJ. Synchronous multicentric osteosarcoma involving mandible and maxillas. International Journal of Oral and Maxillofacial Surgery. 2011;40(4):446-9.
